# Microbiome−mediated crosstalk between T2DM and MASLD: a translational review focused on function

**DOI:** 10.3389/fendo.2025.1677175

**Published:** 2025-11-17

**Authors:** Menghui Jing, Yuanye Jiang

**Affiliations:** 1 Department of Clinical Development, Sunshine Guojian Pharmaceutical (Shanghai) Co. Ltd, Shanghai, China; 2 Department of Gastroenterology, Putuo Hospital, Shanghai University of Traditional Chinese Medicine, Shanghai, China

**Keywords:** gut microbiota, type 2 diabetes mellitus, metabolic dysfunction –associated steatotic liver disease, short chain fatty acids, bile acids/FXR/TGR5 signaling, microbiome biomarkers

## Abstract

Type 2 diabetes mellitus (T2DM) and metabolic dysfunction–associated steatotic liver disease (MASLD) frequently co-occur and aggravate one another through shared pathways of insulin resistance, low-grade inflammation and disordered lipid handling. Framing their interaction through the gut–liver–pancreas axis, this review synthesizes recent progress with a function-first emphasis, moving beyond taxonomic lists to the microbial outputs most consistently linked to dual metabolic–hepatic endpoints. We summarize how short-chain fatty acids (SCFAs), bile acids (BAs), lipopolysaccharide (LPS) and other microbe-associated molecular patterns, branched-chain amino-acid (BCAA) catabolites, trimethylamine N-oxide (TMAO) and endogenous ethanol reach the liver via portal inflow or the enterohepatic BA cycle and act on epithelial, immune and endocrine interfaces, including the farnesoid X receptor (FXR), G-protein–coupled BA receptor 1 (TGR5) and fibroblast growth factor 19/15 signaling. Mechanistic routes—barrier dysfunction and endotoxaemia; SCFA signaling with effects on enteroendocrine tone and substrate flux; BA remodeling that resets hepatic and pancreatic set-points; and nitrogen/choline and ethanol pathways that promote lipotoxic injury—offer biologically coherent explanations for parallel trajectories of hyperglycemia and steatosis/inflammation. We appraise therapeutic modulation spanning diet and fermentable substrates, live biotherapeutics/postbiotics, BA-targeting drugs, fecal microbiota transplantation and metabolic/bariatric surgery, and we outline clinically actionable biomarker opportunities using function-based panels (fermentative capacity, BA transformation, inflammatory ligands, nitrogen/methyl flux) integrated with host metabolites and genetics for diagnosis, risk stratification and response prediction. By advocating standardized reporting, careful control of diet/medications and composite metabolic–hepatic endpoints in prospective trials, this review provides a practical framework to accelerate translation from association to targeted prevention and therapy that improves glycemic control and MASLD activity in parallel.

## 1 Introduction

Type 2 diabetes mellitus (T2DM) and metabolic dysfunction–associated steatotic liver disease (MASLD) frequently coexist and share core pathophysiological features, including systemic and hepatic insulin resistance, low-grade inflammation, and disordered lipid trafficking. MASLD is a newly defined umbrella term that replaces the former “non-alcoholic fatty liver disease (NAFLD),” introduced by a global consensus panel in 2023 to better reflect the underlying metabolic etiology and remove ambiguity surrounding alcohol use (1). Epidemiological and clinical observations indicate that glycemic deterioration and hepatic steatosis/inflammation often progress in parallel and may reinforce one another (2–4). This convergence supports viewing T2DM and MASLD not as isolated entities but as interconnected manifestations along a continuum of metabolic dysfunction. In practical terms, such a perspective encourages integrated endpoints—combining glycemic control with hepatic steatosis, inflammatory activity, and fibrosis risk—rather than siloed disease management (5).

The human gut microbiome—comprising trillions of bacteria, archaea, viruses, and fungi—plays a crucial role in regulating host metabolism. Through fermentation of dietary fibers and processing of amino acids and bile acids, the microbiome generates a wide array of bioactive metabolites. Over the last five years, the gut microbiota has emerged as a mechanistic conduit capable of influencing both glucose homeostasis and liver disease activity via the gut–liver–pancreas axis (6–8). The field has progressively moved beyond lists of differentially abundant taxa to emphasize function-centered outputs. Microbial metabolites and structural components—including short-chain fatty acids (SCFAs), bile-acid (BA) derivatives, lipopolysaccharide (LPS) and other microbe-associated molecular patterns (MAMPs), branched-chain amino acid (BCAA) catabolites, trimethylamine-N-oxide (TMAO), and endogenously produced ethanol/aldehydes—act on intestinal, hepatic, pancreatic, and neural interfaces (9). These signals modulate epithelial barrier tone and innate immune activation; engage BA-sensing receptors such as farnesoid X receptor (FXR) and G-protein–coupled bile-acid receptor 1 (TGR5); and shape enteroendocrine hormone secretion, including glucagon-like peptide-1 (GLP-1), glucose-dependent insulinotropic polypeptide (GIP), and peptide YY (PYY). Collectively, these pathways provide plausible routes by which the same microbial functions can alter hepatic lipid flux, β-cell stress, and systemic insulin sensitivity (10, 11).

**Figure 1:**
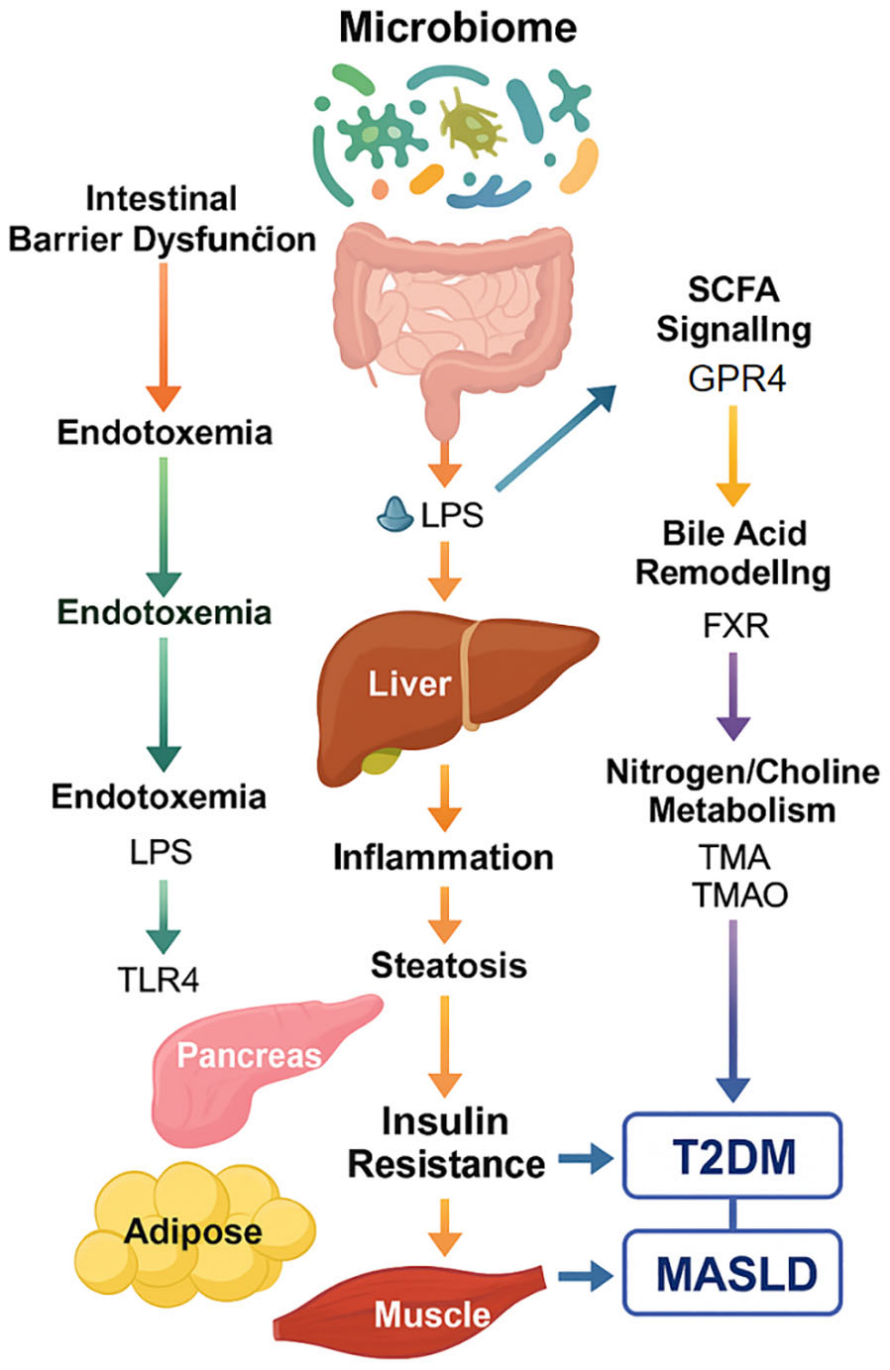
Mechanistic overview of gut microbiome-driven pathways linking T2DM and MASLD.

This review adopts MASLD terminology and concentrates on recent, concept-level progress that is directly relevant to translational researchers and clinicians. We first outline the anatomical conduits and signaling interfaces that enable gut-derived factors to co-regulate glycemic and hepatic endpoints. We then summarize reproducible microbiome features in T2DM and MASLD at the functional level, discuss key mechanistic routes with emerging causal support, and briefly appraise interventions—nutritional strategies, live biotherapeutics/postbiotics, bile-acid–targeting agents, fecal microbiota transplantation, and metabolic surgery—that may deliver dual benefits. We close with a concise synthesis of biomarker opportunities and outstanding gaps to guide future work (**Figure 1**).

This schematic summarizes the five major microbiome-mediated routes—intestinal barrier dysfunction, SCFA signaling, bile acid remodeling, endotoxemia (LPS), and nitrogen/choline metabolism—that collectively influence hepatic inflammation, insulin resistance, and metabolic dysfunction. These interlinked mechanisms serve as potential therapeutic targets and biomarker sources for precision intervention in metabolic-liver disease.

## 2 The gut–liver–pancreas axis: anatomical conduits and signaling interfaces

### 2.1 Portal inflow and enterohepatic cycling as anatomical “fast lanes”

The structural design of the portal circulation channels luminally derived molecules directly from the intestine to the liver, exposing hepatocytes, Kupffer cells (KCs), hepatic stellate cells, and sinusoidal endothelium to high concentrations of dietary catabolites, microbial metabolites, and MAMPs before significant systemic dilution occurs. In parallel, the enterohepatic BA cycle returns microbially transformed bile acids to the liver, providing a second rapid stream of gut-conditioned signals (12, 13). These two conduits operate in tandem: portal inflow delivers SCFAs, ethanol/aldehydes, BCAA catabolites, and LPS that influence hepatic gluconeogenesis, *de novo* lipogenesis, inflammation, and fibrogenesis, while BA recirculation modulates hepatocellular and nuclear receptor signaling that further tunes glucose and lipid handling. Because these inputs are also sensed by the pancreas and integrated through neural circuits, the same gut-derived signals can synchronously affect hepatic steatotic injury and systemic glycaemia, offering a structural explanation for parallel clinical trajectories in T2DM and MASLD (14, 15).

### 2.2 Barrier integrity and innate immune tone as determinants of inflammatory “set-points”

The intestinal barrier—comprising epithelial tight junctions, the mucus layer, and secretory immunoglobulin A—sets the baseline for exposure to luminal content (16). When diet, medications, microbial community shifts, or circadian disruption weaken this barrier, the probability of MAMP translocation increases, with pattern-recognition receptors (PRRs) such as Toll-like receptors and nucleotide-binding oligomerization domain receptors in the liver and adipose tissue sensing these inputs. The resulting low-grade inflammation reinforces hepatic and peripheral insulin resistance and augments susceptibility to lipotoxic injury (17). Conversely, butyrate-rich SCFA profiles generated from fermentable fiber, together with intact mucus dynamics and appropriate epithelial turnover, support tight-junction maintenance and reduce endotoxaemic load (18). The emerging concept is not a binary “leaky gut” but a dynamic barrier tone that fluctuates with diet quality, microbial fermentation, and host behavioral rhythms, thereby modulating the inflammatory set-point that couples T2DM control to MASLD activity.

### 2.3 Bile-acid–receptor signaling as a bidirectional metabolic hub

Gut microbes remodel the BA pool through deconjugation and dehydroxylation, altering ligand availability for FXR and TGR5 along the intestine–liver axis. Activation of intestinal FXR induces fibroblast growth factor 19/15 (FGF19/15), which signals to hepatocytes to restrain BA synthesis and to regulate gluconeogenesis and very-low-density lipoprotein (VLDL) export; TGR5 engagement influences energy expenditure and stimulates GLP-1 release from L-cells (19–21). These mechanisms create a bidirectional hub: BA composition and flow shape microbial niches, while microbial BA transformations reset receptor-level thresholds for hepatic and endocrine metabolism. Therapeutically, this architecture explains why BA-targeting agents and dietary strategies that shift BA dynamics may exert dual effects on glycaemia and MASLD activity, although interindividual variation in BA pools and microbial ecology likely determines both efficacy and safety windows.

### 2.4 Enteroendocrine and neural relays linking nutrient sensing to metabolic control

Enteroendocrine cells (EECs) integrate microbial metabolites and BA signals to modulate secretion of GLP-1, GIP, and PYY, thereby regulating insulin secretion, gastric emptying, appetite, and intestinal motility (22, 23). These hormonal outputs are further integrated with vagal afferents and central circuits that adjust hepatic autonomic tone, influencing hepatic glucose production and peripheral substrate utilization on short time scales. Clinically used agents such as GLP-1 receptor agonists (GLP-1RA) and sodium-glucose co-transporter-2 inhibitors (SGLT2i) primarily act on host targets but secondarily remodel the gut ecosystem through weight loss, altered nutrient flow, and BA changes, creating feedback between pharmacology and microbiome (24–26). Such feedback helps account for instances in which therapies initially developed for diabetes show ancillary benefits on liver fat and inflammation, and it underscores the importance of considering gut signaling when interpreting drug responses across metabolic endpoints.

### 2.5 From taxa to functions: reproducible features with translational value

Across populations and study designs, functional readouts have proven more consistent than single-taxon associations in mapping the microbiome to T2DM and MASLD. Activities related to LPS biosynthesis, bile-salt hydrolase function, SCFA production, BCAA and TMA/TMAO pathways, and endogenous ethanol generation align more robustly with insulin resistance, hepatic lipid accumulation, inflammatory activity, and fibrosis risk. Experimental transfers into gnotobiotic hosts, targeted metabolite supplementation or inhibition, and early human interventional studies—including fecal microbiota transplantation—have begun to move selected observations from correlation toward causality (27). For translation, these functional signatures provide measurable biomarkers and rational targets for postbiotic or pathway-directed interventions designed to improve glycaemia and liver disease activity simultaneously (28).

**Figure 2:**
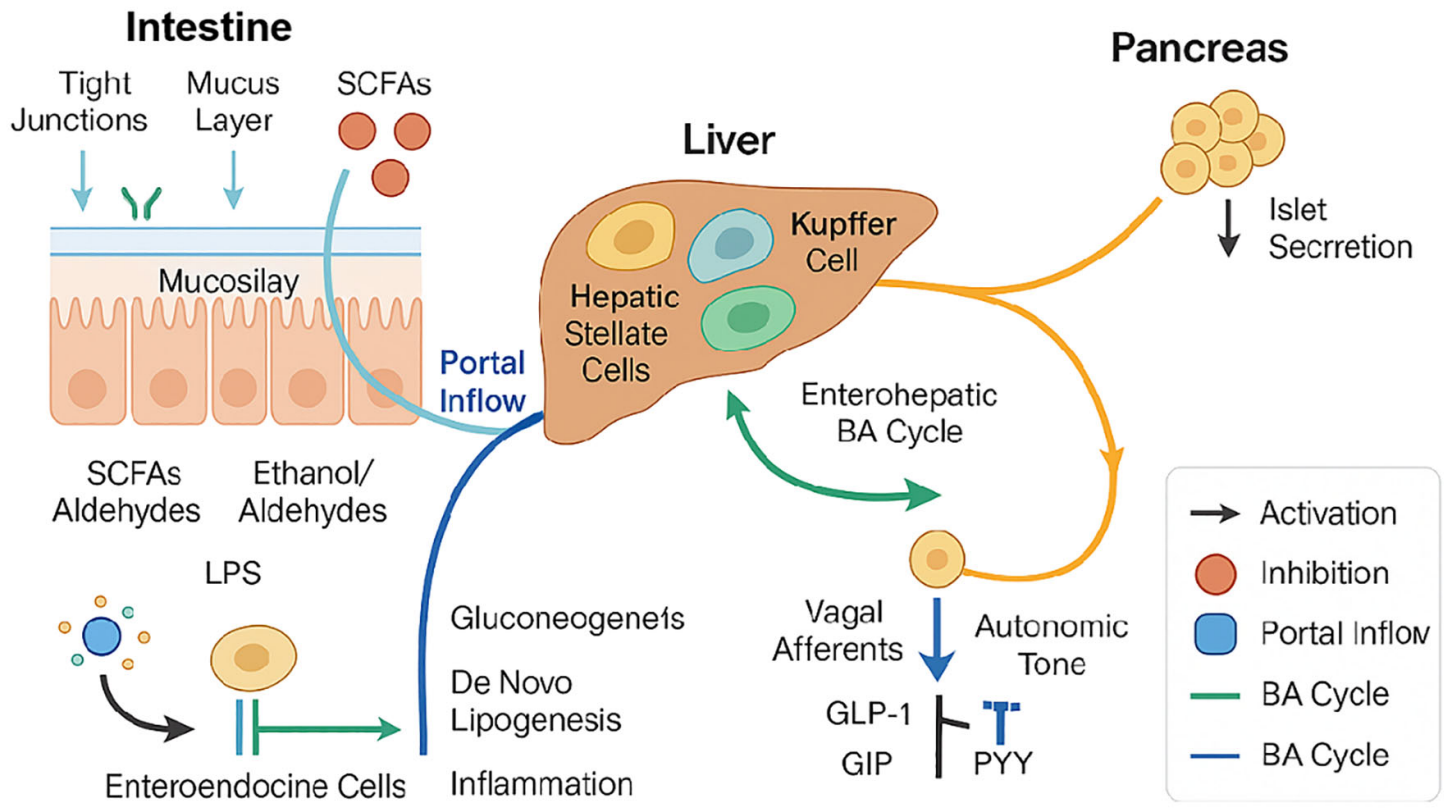
The gut–liver–pancreas axis integrates anatomical conduits (portal inflow and enterohepatic bile−acid cycling) with signaling interfaces.

Key message: Taken together, the portal and biliary conduits provide rapid delivery and amplification of gut-conditioned signals, while barrier/innate immune mechanisms, BA-receptor pathways, and enteroendocrine-neural relays distribute and integrate those signals across liver and pancreatic physiology. Within this framework, functional microbiome features—rather than discrete taxa—map most consistently to dual endpoints relevant to T2DM and MASLD. This synthesis offers a mechanistic basis for why dietary patterns, fiber-derived postbiotics, BA-targeting strategies, metabolic surgery, and some antidiabetic drugs can deliver parallel improvements in glycaemia and liver health. It also points to the need for “cleaner” clinical studies that stratify by BA profiles, diet, and medication use, and that measure functional microbiome outputs alongside standard metabolic outcomes to clarify causality and optimize patient selection (**Figure 2**).

Intestinal barrier integrity—maintained by tight junctions and mucus layer—is disrupted under dysbiosis, allowing microbial products such as SCFAs, ethanol/aldehydes, and LPS to translocate via the portal vein into the liver. These metabolites modulate gluconeogenesis, *de novo* lipogenesis, and hepatic inflammation through activation of hepatic stellate cells and Kupffer cells. Enteroendocrine signaling (e.g., GLP-1, GIP, PYY) impacts both liver metabolism and pancreatic islet hormone secretion via vagal and autonomic pathways. Bile acid metabolism is influenced by gut microbiota and reciprocally shapes enterohepatic signaling loops. Arrows indicate activation (black), inhibition (orange), portal inflow (blue), and bile acid cycle directions (green). This diagram integrates microbiota-derived metabolites and host metabolic responses central to the pathophysiology of T2DM and MASLD.

## 3 Microbiome signatures across type 2 diabetes mellitus and metabolic dysfunction–associated steatotic liver disease

### 3.1 From taxonomy to functions: what current data actually agree on

Across recent cohorts, shotgun metagenomics coupled with targeted or untargeted metabolomics has shifted emphasis from lists of differentially abundant species to function-level readouts that better align with clinical phenotypes (29–32). Community diversity metrics vary with geography and diet, and disease–control separations on beta diversity often diminish after adjustment for lifestyle. By contrast, pathway signatures recur: enrichment of LPS biosynthesis modules and bile salt hydrolase activities; altered capacity for SCFA production—especially butyrate-linked guilds; increased potential for endogenous ethanol generation; re-weighting of BCAA turnover; and formation of trimethylamine (TMA)/trimethylamine-N-oxide (TMAO) (33). These functions correlate more consistently than single taxa with insulin resistance, hepatic lipid accumulation, and inflammatory activity across datasets and remain informative in models that include age and adiposity. This function-first view also improves portability across platforms and pipelines, creating a clearer bridge to mechanistic interpretation and to the design of interventions that target measurable microbial outputs (34, 35).

### 3.2 Shared backbone and disease-specific emphases in T2DM and MASLD

In T2DM, profiles frequently indicate reduced butyrate-generating capacity together with higher representation of inflammatory and oxidative-stress modules, a combination that mirrors systemic low-grade inflammation and impaired insulin signaling (34). Functional features related to BCAA liberation or incomplete catabolism associate with elevated circulating BCAA and insulin-resistance phenotypes, while shifts in bile-acid transformation potential can rebalance engagement of FXR and TGR5, with downstream effects on gluconeogenesis and enteroendocrine output (13, 36).

In MASLD, pathway sets more consistently implicate gut-derived inflammatory and lipogenic drives delivered via the portal vein. Increased capacity for LPS and peptidoglycan biosynthesis, ethanol/aldehyde production, and deconjugation/7α-dehydroxylation of bile acids tracks with steatosis severity and, in several cohorts, with ballooning and fibrosis stage. These transformations are mechanistically coherent: secondary bile-acid shifts alter receptor-level thresholds for hepatic glucose output and very-low-density lipoprotein (VLDL) export, whereas endogenous ethanol perturbs hepatocellular redox and sensitizes stellate cells (37, 38). Pediatric and adult MASLD may diverge in both taxonomic composition and pathway weighting—likely reflecting diet, medication exposure, and developmental physiology—arguing for age-stratified analyses when interpreting signatures (13, 39). Superimposed on these disease-specific emphases is a shared backbone of barrier-relevant and bile-acid–modifying functions that raise inflammatory tone and reset hepatic and endocrine set-points, helping explain the frequent clinical co-expression of T2DM and MASLD (38).

### 3.3 Integration and translation: how to use these signatures

Function-based panels that integrate fermentative capacity (e.g., butyrate pathways), bile-acid transformation potential, inflammatory ligand production, and nitrogen-containing metabolite routes offer a pragmatic scaffold for risk stratification across the T2DM–MASLD continuum. In practice, such panels can support diagnostic adjuncts, enrich trials by selecting participants most likely to benefit from fiber-forward diets, bile-acid–modulating drugs, or incretin-based therapies, and enable response monitoring that pairs microbiome functions with glycemic and hepatic endpoints. Longitudinal interventions provide internal coherence to this approach: weight-loss programs, metabolic surgery, and fermentable-fiber augmentation tend to shift the microbiome toward a higher-butyrate, lower-inflammation state, whereas approved antidiabetic agents such as GLP-1 receptor agonists and sodium-glucose co-transporter-2 inhibitors remodel gut ecology secondarily via weight change, nutrient flow, and bile-acid dynamics, coinciding with improvements in glycaemia and liver fat (31, 32, 40, 41). To enhance interpretability and replication, studies should prospectively capture diet and medication use, pre-specify stratifications (e.g., by bile-acid profile or drug class), and normalize functional features across batches. Embedding these standards in interventional designs will clarify causality, refine patient selection, and accelerate translation from associative signatures to actionable targets that influence both glucose control and liver disease activity.

Key message: Recent research increasingly highlights that function-level microbial signatures—such as SCFA production, LPS biosynthesis, BCAA metabolism, and bile acid transformation—are more consistent and translatable than taxonomic shifts alone. These functional profiles not only align with clinical endpoints like insulin resistance and steatosis but also demonstrate reproducibility across cohorts, improving their utility for biomarker development. Moreover, T2DM and MASLD exhibit both overlapping and distinct microbial functional alterations, suggesting shared mechanisms alongside disease-specific nuances. These insights lay the foundation for precision strategies that leverage microbial functions to support diagnosis, prognosis, and therapeutic selection.

## 4 Mechanistic routes linking microbiome functions to T2DM–MASLD crosstalk

### 4.1 Barrier dysfunction, endotoxaemia, and pattern-recognition signaling

Compromise of the intestinal barrier increases exposure of the liver and adipose tissue to MAMPs such as LPS and peptidoglycan that reach the liver via the portal vein. Engagement of pattern-recognition receptors (PRRs)—notably Toll-like receptors (TLRs) and nucleotide-binding oligomerization domain (NOD) proteins—on Kupffer cells (KCs), hepatocytes, and adipose macrophages activates downstream nuclear factor-κB and interferon pathways, establishing low-grade inflammation that reinforces insulin resistance in liver and peripheral tissues (42). This inflammatory set-point promotes *de novo* lipogenesis (DNL), impairs insulin-mediated suppression of hepatic glucose production, and sensitizes the liver to lipotoxic injury. Barrier tone is dynamic and shaped by diet, circadian behaviors, and microbial fermentation; butyrate-rich SCFA profiles support tight-junction integrity, mucus renewal, and epithelial energy supply, thereby lowering endotoxin exposure (43). Together, these elements provide a causal bridge from gut ecology to simultaneous deterioration of glycaemia and MASLD activity.

### 4.2 SCFAs, epithelial–immune crosstalk, and enteroendocrine control

Microbial fermentation of fermentable fiber yields acetate, propionate, and butyrate, which act locally and systemically. Butyrate fuels colonocytes and inhibits histone deacetylases, reinforcing barrier integrity and tempering inflammatory gene expression (44, 45). Free fatty-acid receptors 2 and 3 (FFAR2/FFAR3; also termed GPR43/GPR41) on enteroendocrine cells sense SCFAs to stimulate gGLP-1 and PYY release, linking luminal fermentation to insulin secretion, gastric emptying, and appetite control (46–48). In the liver, acetate and propionate differentially modulate substrate flux; propionate may restrain gluconeogenesis under specific nutritional contexts, whereas excessive acetate delivery can favor lipogenesis when hepatic insulin signaling is impaired. The net metabolic effect thus depends on the balance of SCFAs, the background diet, and hepatic insulin sensitivity. In aggregate, SCFA-driven improvements in barrier function and enteroendocrine tone provide a coherent route by which fiber-forward diets and postbiotic strategies yield dual benefits for glycaemia and hepatic steatosis/inflammation.

### 4.3 Bile-acid remodeling and endocrine signaling via FXR/TGR5–FGF19/15

Gut microbes remodel the bile-acid (BA) pool through deconjugation and dehydroxylation, altering ligand availability for FXR and TGR5 along the intestine–liver axis (49). Activation of intestinal FXR induces fibroblast growth factor 19/15 (FGF19/15), which signals to hepatocytes to repress BA synthesis and to recalibrate gluconeogenesis and VLDL production; TGR5 engagement increases energy expenditure and stimulates GLP-1 secretion from L-cells (20). Because BA composition also selects for specific microbes, these circuits form a bidirectional hub in which microbial BA transformations can reset hepatic and endocrine thresholds for glucose and lipid handling, while host factors (diet, weight loss, medications) feedback to reshape microbial niches. Clinically, this architecture explains why BA-targeting interventions and therapies that secondarily shift BA dynamics—such as GLP-1RA, SGLT2i, and metabolic surgery—can produce parallel improvements in glycemic control and MASLD activity in appropriately selected patients (50, 51).

### 4.4 Nitrogen and choline pathways, endogenous ethanol, and hepatic injury

Microbiome-linked branched-chain amino acid (BCAA) liberation and incomplete catabolism associate with elevated circulating BCAA and impaired insulin signaling, providing a plausible route from proteolytic fermentation to systemic insulin resistance. In the nitrogen–methyl axis, microbial conversion of dietary choline and carnitine to trimethylamine (TMA) and host oxidation to trimethylamine-N-oxide (TMAO) have been linked to cardiometabolic risk and may influence hepatic lipid trafficking and inflammation; simultaneously, microbial consumption of choline can limit hepatic phosphatidylcholine availability, constraining VLDL assembly and promoting steatosis (52, 53). A distinct set of taxa generate endogenous ethanol and aldehydes, which reach the liver via the portal vein, perturb mitochondrial redox and lipid peroxidation, and activate stellate cells—changes that align with progression from simple steatosis to steatohepatitis and fibrosis. These nitrogen, methyl, and ethanol pathways thus provide convergent mechanisms by which gut metabolism can aggravate both T2DM phenotypes and MASLD severity, particularly when combined with high-fat, low-fiber diets that diminish SCFA-mediated protection (54, 55).

Across these mechanisms, a coherent picture emerges: barrier-conditioned MAMP influx sets inflammatory tone; SCFA signaling couples fermentation to epithelial fitness and enteroendocrine control; bile-acid remodeling establishes an endocrine hub that tunes hepatic and pancreatic metabolism; and BCAA/TMAO/ethanol pathways add organ-specific pressures that intensify hepatic lipid accumulation and systemic insulin resistance. This integrated view clarifies why interventions that increase fermentable substrates, stabilize BA signaling, or attenuate nitrogen/ethanol fluxes can deliver dual metabolic benefits, and it motivates clinical trials that stratify participants by functional microbiome profiles and measure these outputs alongside standard glycemic and hepatic endpoints.

Key message: The interplay between microbial functions and host metabolism is mediated through several coherent mechanistic routes. Disruption of intestinal barrier integrity leads to endotoxaemia and chronic inflammation; SCFAs regulate epithelial health and enteroendocrine signaling; bile acid remodeling modulates receptor-mediated metabolic control; and nitrogen/choline pathways and microbial ethanol production promote hepatic injury. These integrated axes explain how gut microbial metabolism influences both glycemic regulation and liver pathology. Understanding these mechanisms provides a biological rationale for interventions that target specific microbial functions to achieve dual metabolic and hepatic benefits.

## 5 Therapeutic modulation of the microbiome

### 5.1 Diet patterns and fermentable substrates as first-line levers

Diet remains the most controllable driver of microbiome function and the most scalable lever for dual endpoints inT2DM and MASLD. Patterns that emphasize minimally processed plant foods—such as Mediterranean-style or targeted high-fiber diets—consistently increase short-chain fatty acid (SCFA) production capacity, particularly butyrate, while reducing the representation of inflammatory ligand pathways such as lipopolysaccharide (LPS) biosynthesis. In practical terms, greater intake of fermentable fibers (e.g., inulin, resistant starches, β-glucans) strengthens epithelial barrier tone, enhances GLP-1 and PYYsignaling via FFAR2/FFAR3, and attenuates hepatic lipogenesis by lowering endotoxin exposure through the portal vein (56–58). Weight loss—whether achieved through caloric restriction, time-restricted eating, or macronutrient rebalancing—amplifies these effects by reducing hepatic substrate oversupply and improving peripheral insulin sensitivity. These shifts often co-occur with favorable BA remodeling (e.g., reduced hydrophobic secondary BA load), providing a mechanistic rationale for parallel improvements in glycaemia and liver fat. Implementation should pair clear fiber targets with pragmatic food lists and brief behavior support; measuring diet quality and medication use alongside outcomes improves interpretability.

### 5.2 Live biotherapeutics, consortia, and postbiotics: when to add and what to expect

Single-strain probiotics have shown variable effects on glycaemia and hepatic indices, reflecting strain heterogeneity and short exposure windows. Current evidence is more congruent for defined consortia that restore fermentative guilds (including butyrate producers) or Akkermansia-enriched formulations that improve mucus dynamics; these products tend to produce modest, directionally favorable changes in insulin sensitivity and hepatic steatosis markers when layered on diet and weight management (59). Postbiotics—purified microbial metabolites or cell-free preparations—offer a more standardizable way to deliver mechanism-specific benefits (e.g., butyrate donors, propionate esters, or bile-salt hydrolase inhibitors/agonists), with fewer colonization uncertainties (60). Fecal microbiota transplantation (FMT) provides the strongest proof-of-principle for causality but has heterogeneous metabolic responses and non-trivial regulatory and safety considerations. A practical approach is stepwise: optimize diet and weight first; consider an adjunct biotherapeutic/postbiotic where functional deficits are evident (e.g., low SCFA production capacity or unfavorable BA profile); and reserve FMT for research settings or highly selected cases with rigorous donor screening and outcome monitoring (61).

### 5.3 Pharmacologic modulation: bile-acid–targeting agents and antidiabetic therapies with microbiome feedback

The FXR–fibroblast growth factor 19/15 (FGF19/15) axis and TGR5 constitute a tractable endocrine hub (62). Agents that modulate these pathways can shift hepatic glucose output, VLDL export, and inflammatory tone while secondarily remodeling the gut ecosystem through BA composition and flow. In parallel, widely used antidiabetic therapies—GLP-1RAand SGLT2i—act primarily on host targets but feedback on the microbiome by altering nutrient transit, BA pools, and energy balance, changes that align with observed reductions in liver fat and transaminase levels in subsets of patients (63, 64). These bidirectional effects recommend a function-aware lens for pharmacotherapy: baseline BA profiles and fermentative capacity may explain part of the between-patient variability in hepatic responses to GLP-1RA/SGLT2i or to BA-targeted drugs, suggesting a path to trial enrichment and patient selection using microbiome functions rather than taxa (65, 66). Routine antibiotic use to “reset” the microbiome is not supported for metabolic indications given transient effects and off-target risks; if antibiotics are unavoidable, documenting timing and class is important when interpreting metabolic outcomes.

### 5.4 Metabolic surgery as a systems-level reset

Metabolic/bariatric procedures (e.g., Roux-en-Y gastric bypass, sleeve gastrectomy) produce large, durable weight loss and rapid glycemic improvement, accompanied by profound shifts in microbiome function and BA signaling that are partly weight-loss independent (67). Post-operative increases in SCFA-linked pathways, altered BA pools that favor FXR/TGR5 signaling conducive to GLP-1 release, and normalization of inflammatory ligand signatures are well-described and map onto reductions in hepatic steatosis and fibrosis risk markers (10). These findings highlight the mechanistic plasticity of the gut–liver–pancreas axis and reinforce the idea that diet, drugs, and biotherapeutics can be combined to approximate—on a smaller scale—the multi-pathway benefits of surgery in patients who do not meet surgical criteria. A comparative overview of how key interventions—including dietary strategies, pharmacological agents, bariatric surgery, and FMT—modulate microbiome-driven pathways and impact dual metabolic-hepatic endpoints is summarized in **Table 1** and **Supplementary Table S1**.

**Table 1:** Clinical studies across T2DM–MASLD showing dual metabolic–hepatic endpoints.

Category	Intervention	Design/Population/N/Duration	Glycemic outcome	Hepatic outcome
Diet (68) (Prebiotic)	Resistant starch (RS2) 40 g/day	Randomized, placebo-controlled; NAFLD/MASLD; n=200; 4 mo	Modest improvement in insulin/glycemic indices	↓ IHTG ~30–40%; ↓ ALT (partly weight-independent)
Pharmacologic (69) (GIP/GLP-1 RA)	Tirzepatide 5/10/15 mg weekly	Phase 2 RCT; MASH F2–F3; n=190; 52 wk	↓ HbA1c; ↓ weight	MASH resolution 51–62% vs 13%; fibrosis ≥1-stage 51–55% vs 30%
Pharmacologic (41) (GLP-1 RA)	Semaglutide 2.4 mg weekly	Phase 3 RCT; MASH F2–F3; ~n=800; 72 wk	↓ HbA1c; ↓ weight	Higher MASH resolution and fibrosis improvement vs placebo
Pharmacologic (40) (SGLT2 inhibitor)	Empagliflozin 10 mg daily	RCT; non-diabetic MASLD; n=98; 52 wk	Neutral (non-diabetic)	↓ MRI-PDFF vs placebo (Δ ≈ −1.1%)
Metabolic surgery (70)	RYGB/Sleeve gastrectomy	Prospective & retrospective cohorts; biopsy-proven NASH; n=180 & n=1158; up to 10 yr	Sustained improvements	NASH resolution ~84% at 5 yr; fewer long-term liver events
Microbiota transfer (71)	Lean-donor FMT (capsules)	Double-blind RCT; T2DM; ~n=60; 12 wk	No durable HbA1c/ISI benefit	Not primary/insufficient imaging

RCT, randomized controlled trial; MASLD, metabolic dysfunction–associated steatotic liver disease; MASH, metabolic dysfunction–associated steatohepatitis; HbA1c, glycated hemoglobin; MRI-PDFF, magnetic resonance imaging–proton density fat fraction; IHTG, intrahepatic triglyceride; ALT, alanine aminotransferase; RYGB, Roux-en-Y gastric bypass; FMT, fecal microbiota transplantation.

↓ downregulation.

### 5.5 Evidence and challenges: conflicting findings and reproducibility issues

While many studies report beneficial effects of probiotics, synbiotics, and FMT on metabolic parameters and hepatic steatosis, the findings are not uniformly consistent. For example, differing outcomes have been observed depending on the donor microbiota used in FMT, participant baseline microbiome structure, duration of intervention, and concomitant diet or medication (72). In clinical trials and human RCTs, some probiotic formulations show significant improvements in liver enzymes or hepatic fat, whereas others fail to demonstrate benefit, possibly due to small sample size, short follow-up, strain specificity, or heterogeneity in endpoints (73). Furthermore, multi−omic microbiome studies frequently report inconsistent associations when repeated across different cohorts, likely reflecting variability in sequencing platforms, bioinformatics pipelines, population genetics, geography, diet, and other host/environmental confounders (74). Safety concerns and long−term effects, especially for FMT (such as unintended colonization of microbes in non−native niches, or risk of pathogen transfer), remain under−studied. Addressing these challenges will require larger, multicenter RCTs, harmonized protocols, longer follow−ups, and pre−registered analysis plans to enhance reproducibility and facilitate translation.

Key message: Modulating the gut microbiome through diet, therapeutics, and surgical interventions offers a promising route to address both T2DM and MASLD. Diets rich in fermentable fibers enhance SCFA profiles and barrier tone, while select live biotherapeutics and postbiotics can restore functional deficits in fermentation or bile acid modulation. Pharmacologic agents, including GLP1RAs and SGLT2is, indirectly reshape the microbiome via host-mediated pathways, creating bidirectional feedback loops. Metabolic surgery exerts profound and durable shifts in microbiome-derived functions and bile acid profiles. Collectively, these strategies highlight the potential of microbiome-directed therapies as adjuncts or amplifiers of conventional metabolic interventions.

## 6 Microbiome biomarkers in T2DM and MASLD: clinical translation and future directions

### 6.1 Function−based panels for diagnosis and risk stratification

The most credible case for clinical microbiome biomarkers now lies in function-level panels paired with host metabolites. In T2DM, large prospective data show that baseline shotgun metagenomic profiles predict incident disease independent of conventional risk factors: in a Finnish cohort of 5,572 adults followed a median ~15 years, metagenomic features associated with future T2DM and validated across subcohorts, supporting feasibility for population-level risk enrichment (32). In MASLD, recent multi-center analyses emphasize functional signatures (e.g., lipopolysaccharide (LPS) biosynthesis, bile-salt hydrolase and 7α-dehydroxylation capacity, endogenous ethanol pathways) that align with steatosis activity and fibrosis staging more reliably than single taxa; a 2024 study reported robust, disease-specific signatures with improved cross-cohort portability, reinforcing the shift from organism lists to pathway readouts for non-invasive detection and staging (31). For liver disease specifically, non-invasive biomarker frameworks such as the NIMBLE project (focused on NAFLD/MASH biomarker qualification) provide a template for evaluating add-on value of microbiome functions alongside established non-invasive tests (imaging and serum panels), highlighting where further validation is required before qualification (75, 76).

### 6.2 Predicting and monitoring therapeutic response

A pragmatic near-term use case is response prediction. Baseline fermentative capacity (particularly butyrate-linked pathways) and bile-acid (BA) transformation potential often track with improvements in glycaemia and liver fat during weight-loss diets or fiber-forward interventions; multiple diet trials and systematic syntheses suggest that the pretreatment microbiome can forecast weight-loss and hepatic fat responses, although effect sizes vary with design and adherence (68, 77). In pharmacotherapy, widely used antidiabetic agents—GLP-1RA and SGLT2i—show secondary microbiome remodeling after initiation, and emerging human data indicate that baseline fecal features can predict glycemic response to these drugs, suggesting a path to function-aware patient selection and monitoring (78). For metabolic/bariatric surgery, longitudinal cohorts link resolution of NAFLD/MASLD to post-operative shifts in gut microbial functions and plasma BA species, supporting the concept that combining stool metagenomics with BA profiles could serve as a monitoring tool when imaging or biopsy is impractical. Finally, fecal microbiota transplantation (FMT) continues to provide proof-of-principle for causality; randomized and controlled metabolic-syndrome/T2DM studies report directionally favorable changes in insulin sensitivity and SCFA-producing guilds, albeit with heterogeneous durability—underscoring the need for better recipient stratification and standardized endpoints (70, 79, 80).

### 6.3 Integrating microbiome functions with host multi−omics

The most informative models combine microbiome functions with host multi-omics. Recent work integrating polygenic risk scores with gut metagenomics demonstrates that microbiome-derived risk can match or complement traditional clinical predictors for cardiometabolic diseases, including T2DM, suggesting additive value beyond age, blood pressure and lipids (29). In MASLD, multi-omic subtyping has identified biologically distinct forms of severe disease using genomics, transcriptomics, proteomics and metabolomics, a framework that can incorporate microbial functions and BA species to refine fibrosis risk and treatment targeting (81). Integration with host genetics (e.g., PNPLA3, TM6SF2) and dietary exposures is increasingly emphasized; contemporary analyses illustrate how inherited risk and nutrition interact with microbial pathways to shape liver fat and inflammation, an approach that naturally extends to function-level microbial markers (82).

Increasing evidence suggests that baseline microbiome functional profiles, host genetic variants, and dietary context jointly determine individual responses to microbiome−targeted therapies (29). For example, the capacity for butyrate or bile acid transformation at baseline may stratify likelihood of response to fermentable fiber, GLP−1 receptor agonists, or FXR/TGR5−targeted drugs. Host genetic polymorphisms—such as in PNPLA3, TM6SF2, and HSD17B13—modulate hepatic lipid metabolism and inflammatory tone, and may interact with microbial metabolite signaling to influence therapeutic efficacy (83). Dietary patterns, particularly habitual fiber intake or choline burden, further shape the ecological context in which interventions act. As such, precision microbiome modulation will require integrated assessment of microbial functions, host genetics, and modifiable exposures (84, 85). Future trials should incorporate stratification strategies based on these variables, enabling more targeted, reproducible, and patient−centered intervention approaches.

### 6.4 Standardization and clinical implementation

Translational credibility depends on pre-analytical rigor and transparent reporting. The STORMS guideline (Strengthening the Organization and Reporting of Microbiome Studies) is now the de facto reporting standard for human microbiome research and should be paired with CONSORT/STARD/TRIPOD as appropriate (30). Studies should prospectively capture diet and medication use, standardize sample collection and storage, and report batch correction and functional normalization strategies. On the analytics side, recent cross-study evaluations show that without strict separation of training/validation data and without leakage control, claimed performance often collapses when models face external cohorts; interpretable or sparse machine-learning pipelines with cross-cohort validation are therefore preferred for clinical translation. For clinical labs, near-term assays should favor targeted pathway panels (e.g., butyrate/propionate modules, LPS biosynthesis, BA-modifying enzymes) and paired host metabolites (stool/plasma SCFAs, BA species), reported as risk strata or response probabilities rather than raw abundances—facilitating integration with existing non-invasive tests and electronic health records (86).

### 6.5 Regulatory and ethical considerations for microbiome−based therapeutics

The U.S. Food and Drug Administration (FDA) has established precedents for live microbiota products, approving REBYOTA (fecal microbiota, live-jslm) in November 2022 and VOWST (oral fecal microbiota spores) in April 2023—both for recurrent Clostridioides difficile—and posting specific guidance and safety communications for fecal microbiota products. These actions delineate expectations for donor screening, manufacturing quality, and post-marketing surveillance that will inform future metabolic indications (87). For T2DM/MASLD applications, ethics considerations include informed consent for stool-derived data, data privacy for sequencing-based diagnostics, and equitable access to diet-centered or biotherapeutic interventions. Any exploration of FMT for metabolic endpoints should remain within regulated trials that adhere to FDA advisory committee recommendations and safety monitoring.

Recent clinical milestones have significantly advanced the therapeutic landscape of MASLD. Notably, Resmetirom (Rezdiffra), a selective thyroid hormone receptor-β (THR−β) agonist, became the first FDA-approved drug for MASH in 2024, demonstrating robust efficacy in reducing hepatic steatosis and fibrosis progression across phase 3 trials (88). Concurrently, fibroblast growth factor 21 (FGF21) analogues such as pegozafermin, efruxifermin, and BIO89–100 have shown dual benefit in improving both metabolic parameters (e.g., glycemia, triglycerides, insulin sensitivity) and hepatic histology (steatosis, ballooning, inflammation) (89–91).

These emerging agents provide critical context for microbiome-targeted strategies. For example, Resmetirom modulates bile acid composition and FXR–FGF19 signaling, while FGF21 analogues influence hepatic lipid oxidation, adipose lipolysis, and energy expenditure—pathways that are increasingly recognized to interact with gut microbiota-derived metabolites. Future microbiome-based interventions may be layered onto or used to stratify responses to these agents, particularly via profiling of bile acid–modifying bacteria, SCFA fermentation potential, and inflammation-linked microbial signatures.

As MASLD therapeutics shift toward precision endpoints and combinatorial approaches, integrating function-based microbiome metrics with emerging drug mechanisms offers a pathway toward more personalized and effective treatment strategies.

### 6.6 Challenges, knowledge gaps and trial design priorities

Three methodological gaps dominate. First, causality: despite encouraging Mendelian randomization (MR) signals linking specific microbial taxa or pathways to T2DM sub-phenotypes and to trimethylamine-N-oxide (TMAO) biology, instruments remain weak and heterogeneous; triangulation with longitudinal interventions, gnotobiotic transfers, and pathway-targeted postbiotics is required (34, 92). Second, confounding: diet quality, alcohol exposure, energy balance, antibiotics, metformin, proton-pump inhibitors, and statins strongly shape microbiome functions and must be measured and, where possible, controlled. Consensus reports in diabetes now explicitly recommend capturing these covariates when interpreting microbiome data in clinical studies (41). Third, endpoints and generalizability: microbiome-guided strategies should be tested against composite outcomes that reflect the dual goal (glycaemia + liver fat/inflammation/fibrosis). Recent hepatology trials illustrate feasible histologic and non-invasive endpoints (e.g., resolution of metabolic dysfunction–associated steatohepatitis and changes in fibrosis stage) and show that incretin-based agents can deliver parallel benefits—a clinical context in which function-level microbiome markers may help enrich responders and interpret heterogeneity (69, 93).

Design-wise, we advocate pre-registered, multi-arm studies that (i) stratify by baseline BA profile and fermentative capacity; (ii) incorporate standardized diet/medication reporting and STORMS adherence; (iii) measure microbiome functions + host metabolites at baseline/early-response/maintenance; and (iv) use adaptive enrichment to prospectively test whether function-based strata increase effect sizes for diets, postbiotics, BA-targeted agents, or combination regimens.

### 6.7 Clinical integration framework

**Figure 3:**
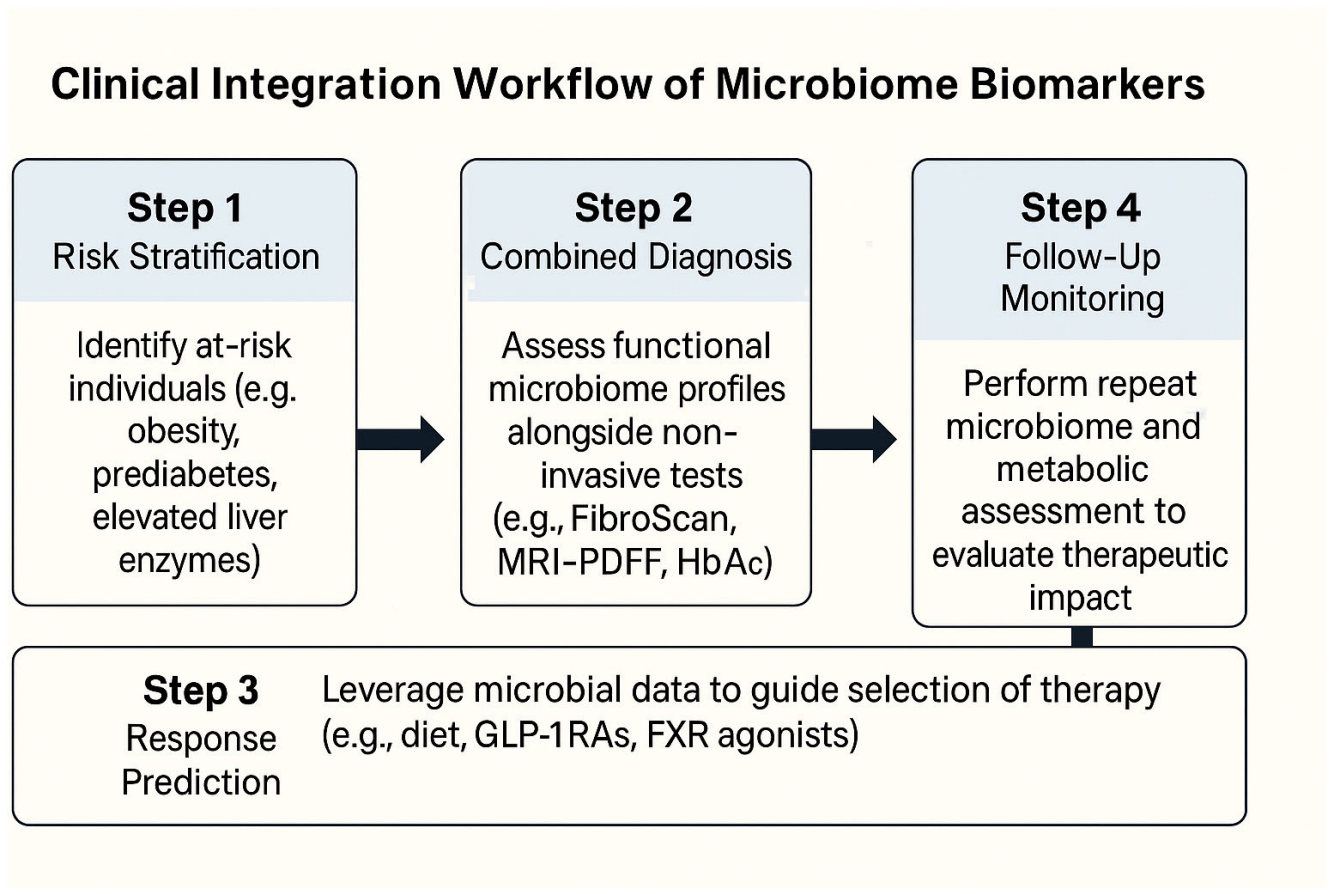
Clinical integration workflow of microbiome biomarkers in T2DM–MASLD comorbidity.

To translate microbiome-based diagnostics into routine care, a simplified, stepwise framework can assist clinicians in selecting, interpreting, and applying microbiome-derived information. First, functional microbiome panels—targeting SCFA production, bile acid transformation, LPS biosynthesis, and nitrogen metabolism—can be deployed in at-risk populations (e.g., patients with obesity, prediabetes, or elevated liver enzymes) to stratify risk of dual metabolic-hepatic progression. Second, combining stool-based microbial functions with existing non-invasive tools (e.g., FibroScan, MRI-PDFF, liver enzyme panels, HbA1c) enhances diagnostic precision, particularly when fibrosis risk or therapeutic escalation is being considered. Third, in pharmacologic or dietary interventions, baseline microbial capacity (e.g., butyrate production or BA profiles) may help predict response and guide selection of GLP-1RAs, SGLT2i, BA-modulating drugs, or high-fiber dietary regimens. Finally, for follow-up, repeated functional testing may allow clinicians to monitor therapeutic impact alongside hepatic and glycemic endpoints, especially in settings where imaging or biopsy is impractical. Embedding microbiome-derived readouts into electronic health records and clinical decision support tools will further facilitate real-world uptake. To illustrate how microbiome-based biomarkers can be operationalized in clinical practice, we propose a simplified integration workflow (**Figure 3**), outlining the translational path from risk stratification to therapeutic monitoring and digital decision support.

A proposed stepwise clinical framework for incorporating gut microbiome functional readouts into patient care pathways. Risk stratification identifies individuals with obesity, prediabetes, or MASLD. Stool-based metagenomic or metabolomic profiling assesses microbial modules such as SCFA production, bile acid transformation, LPS load, and nitrogen metabolism. These are integrated with clinical diagnostics—including FibroScan, MRI-PDFF, HbA1c, and liver enzymes—to support stratified treatment decisions. Personalized interventions (e.g., GLP-1 receptor agonists, SGLT2 inhibitors, targeted diets) are guided by microbiome signatures. Therapeutic monitoring involves functional re-testing and tracking of metabolic endpoints. Decision support systems may embed microbiome-based algorithms into electronic health records (EHRs) to enhance clinical workflow.

### 6.8 Challenges in clinical translation of microbiome-based biomarkers

Despite the promising prospects of functional microbiome-based biomarkers in metabolic diseases, their clinical translation faces significant hurdles. From a technological standpoint, challenges include the lack of standardized protocols for functional metagenomic and metabolomic analyses, as well as inconsistency in sequencing platforms and downstream bioinformatics pipelines. These discrepancies hamper reproducibility and cross-cohort comparability, ultimately limiting the generalizability of findings (94–96).

Biologically, the gut microbiome exhibits high inter-individual variability shaped by factors such as age, sex, ethnicity, host genetics, and comorbid conditions. Moreover, extrinsic influences like diet, medications (e.g., metformin, antibiotics), and environmental exposures introduce additional noise that may obscure true disease associations and confound biomarker performance (97, 98). These factors create significant barriers to establishing robust, disease-specific functional signatures with diagnostic or prognostic utility.

Emerging harmonization efforts—such as the STORMS reporting guidelines for microbiome research—are a critical step toward improving methodological transparency and data integration across studies. Furthermore, large-scale, longitudinal, and multi-ethnic cohort studies are urgently needed to validate candidate biomarkers in real-world settings and assess their predictive accuracy across diverse populations (99, 100). Together, addressing these technical and biological challenges will be essential to realize the clinical potential of microbiome-derived biomarkers in T2DM and MASLD.

Key message: Function-based microbiome biomarkers represent a clinically relevant step forward in the diagnosis, monitoring, and treatment of T2DM and MASLD. When integrated with host metabolites and multi-omics platforms, these signatures can predict disease risk, stratify patients for targeted interventions, and monitor therapeutic responses with improved precision. However, successful translation requires methodological rigor, adherence to reporting standards like STORMS, and thoughtful trial design that incorporates microbiome function as both predictor and endpoint. Regulatory frameworks and ethical considerations will be crucial as microbiome-based diagnostics and therapeutics move toward clinical adoption. The future of precision medicine in metabolic diseases will increasingly rely on functional microbiome readouts aligned with host physiology.

## 7 Discussion

T2DM and MASLD are now increasingly understood as interlinked pathophysiological manifestations within a shared metabolic framework. Central to this interaction is the gut–liver–pancreas axis, where gut microbiota-derived functional outputs—such as SCFAs, bile acid derivatives, LPS, BCAA catabolites, TMAO, and endogenous ethanol—play multifaceted roles in modulating host metabolism and immunity.

These metabolites engage host pathways through diverse routes: SCFAs signal via G-protein coupled receptors (e.g., GPR41/43) to regulate gluconeogenesis and lipolysis; secondary bile acids activate nuclear receptors (e.g., FXR, TGR5) affecting lipid metabolism and inflammation; LPS drives hepatic inflammation through TLR4-mediated Kupffer cell activation; TMAO perturbs insulin signaling via oxidative stress and vascular inflammation; and microbe-derived ethanol alters redox balance in hepatocytes. Together, these mechanisms contribute to systemic insulin resistance, hepatic steatosis, inflammation, and fibrosis.

Importantly, functional microbiome signatures show stronger correlations with clinical phenotypes than taxonomic profiles, and have demonstrated responsiveness to dietary interventions, pharmacotherapy, and metabolic surgery—supporting their role in mediating dual improvements in glycemic and hepatic outcomes.

To translate these insights, future studies should prioritize function-based biomarker integration with non-invasive fibrosis scoring and glycemic indices, stratify participants by fermentative and bile acid metabolic capacity, and incorporate standardized dietary and medication metadata. Interventions should target composite T2DM–MASLD endpoints and embed longitudinal microbiome–metabolome monitoring to infer causality and enhance precision in patient selection. With rigorous design, harmonized reporting, and attention to reproducibility and equity, a function-centered microbiome approach offers a promising avenue for co-managing metabolic and hepatic disorders.

In summary, this review highlights the emerging role of microbial functional signatures in shaping the pathophysiology and clinical trajectory of both T2DM and MASLD. Mechanistically, gut-derived metabolites influence host metabolism through endocrine, immune, and enterohepatic pathways. Integrating these insights into clinical practice requires robust biomarker validation, stratified trial design, and standardized metadata capture. A precision-microbiome approach has the potential to transform how we assess and intervene in metabolic-liver comorbidity.
