# Supplementary material for: Microbiome−mediated crosstalk between T2DM and MASLD: a translational review focused on function
Source: Front Endocrinol (Lausanne). 2025 Nov 17;16:1677175. doi: 10.3389/fendo.2025.1677175 (PMC12665569; doi:10.3389/fendo.2025.1677175)
Supplement: Supplementary file 1 [file Table1.docx]

Table S1. Microbiome Functional Readouts

| **Intervention** | | **Platform** | **Functional signals** | **Host interface(s)** | **Notes** |
| --- | --- | --- | --- | --- | --- |
| Resistant starch (RS2) 40 g/day | | Shotgun metagenomics ± targeted metabolomics | ↑ SCFA/fermentative capacity; ↓ LPS/endotoxaemia markers; compositional shift (e.g., B. stercoris↓) | FFAR2/3 (enteroendocrine); barrier tone | Liver fat reduction partly independent of weight change; aligns with function-first mechanism |
| Tirzepatide 5/10/15 mg weekly | not collected | | Likely BA pool/nutrient flow shifts secondary to weight/incretin effects | FXR/TGR5; incretin axis | Histology endpoints improved; microbiome data pending |
| Semaglutide 2.4 mg weekly^[72]^ | not collected | | Likely BA/nutrient transit changes; indirect microbiome effects | FXR/TGR5; incretin axis | Phase 3 histology benefits; function readouts not in primary report |
| Empagliflozin 10 mg daily | not collected | | Weight and glycosuria effects may secondarily shift BA/SCFA profiles | BA signalling (indirect) | Modest MRI-PDFF reduction; mechanistic microbiome data limited |
| Metabolic surgery (RYGB/SG) | Stool metagenomics; plasma bile acids | | ↑ SCFA pathways; BA pool remodelling (favourable FXR/TGR5 tone); ↓ inflammatory ligand signatures | FXR/TGR5; barrier/PRR | Weight-loss dependent and independent components |
| Lean‑donor FMT (capsules) | Strain-resolved metagenomics | | Transient donor engraftment; inconsistent functional shifts | Varies by donor; limited durability | No durable glycaemic benefit in T2D trials to date |

**Abbreviations:** BA, bile acids; PRR, pattern‑recognition receptor; SCFA, short‑chain fatty acids; RYGB, Roux‑en‑Y gastric bypass; SG, sleeve gastrectomy; T2D, type 2 diabetes.
